# INFLECTION POINTS IN HEARING DETERIORATION: CLINICAL CHARACTERISTICS OF NIHL FROM STEADY-STATE NOISE EXPOSURE

**DOI:** 10.13075/ijomeh.1896.02502

**Published:** 2025

**Authors:** Boya Fan, Han Wang, Gang Wang, Gang Liu, Xiaoli Zhang, Wei Wu, Yulin Kang

**Affiliations:** 1 Peking University Third Hospital, Department of Otolaryngology Head and Neck Surgery, Beijing, China; 2 The Ninth Medical Center of PLA General Hospital, Department of Otolaryngology Head and Neck Surgery, Beijing, China; 3 State Environmental Protection Key Laboratory of Environmental Sense Organ Stress and Health, Beijing, China; 4 Peking University, Department of Occupational and Environmental Health Sciences, School of Public Health, Beijing, China; 5 Chinese Research Academy of Environmental Sciences, Institute of Environmental Information, Beijing, China

**Keywords:** noise-induced hearing loss, clinical characteristics, time inflection point, pure tone audiometry, distorted product otoacoustic emissions, steady-state noise

## Abstract

**Objectives:**

To explore the clinical characteristics of noise-induced hearing loss (NIHL) caused by long-term exposure to steady-state noise and find a possible inflection point time leading to hearing deterioration.

**Material and Methods:**

Subjects exposed to steady-state noise were selected as the noise-exposed group and matched with a control group of individuals not exposed to noise. Both groups underwent pure-tone audiometry (PTA) and distortion product otoacoustic emissions (DPOAE), and their hearing conditions were analyzed. The time inflection point with the most significant disparities in NIHL between early and late exposure was evaluated. The noise-exposed subjects were divided into 2 groups based on cumulative exposure time: the early exposure group (group A) and the late exposure group (group B). Retrospective analyses of clinical characteristics of hearing loss were conducted.

**Results:**

The noise-exposed group exhibited significantly higher hearing thresholds and reduced otoacoustic emissions compared to the control group, with high-frequency hearing loss being the most prominent. The most significant disparity in high-frequency hearing loss in PTA was observed before and after 5 years of cumulative steady-state noise exposure. Among the 78 noise-exposed subjects, 37 were in group A (≤5 years) and 41 in group B (>5 years). In DPOAE, the most significant disparity occurred before and after 4 years of exposure, with 33 subjects in group A (≤4 years) and 45 in group B (>4 years). Distortion product otoacoustic emissions identified the time inflection point of significant hearing deterioration 1 year earlier than PTA.

**Conclusions:**

Hearing loss caused by long-term exposure to steady-state noise showed evident deterioration after 4–5 years. The DPOAE can illustrate the inflection point of hearing deterioration 1 year earlier than PTA.

## Highlights

- Steady-state noise exposure causes significant hearing loss after 4–5 years.
- Distortion product otoacoustic emissions (DPOAE) detects hearing deterioration 1 year earlier than pure tone audiometry.
- High-frequency hearing loss is most significant after 5 years of noise exposure.

## INTRODUCTION

Hearing loss often occurs in daily work and life. In 2018, the World Health Organization (WHO) estimated that 460 million people worldwide have a hearing impairment. By 2030, this number will increase to almost 630 million; by 2050, >900 million people will have hearing loss to some extent [1]. For occupational groups, prolonged exposure to high-intensity noise is a major cause of noise-induced hearing loss (NIHL), one of the most common occupational health issues. Noise-induced hearing loss has always been a main occupational health problem [2,3]. Studies suggest that exposure to noise levels >89 dB for >5 h/week can lead to permanent hearing damage [4]. This highlights the importance of understanding cumulative noise exposure and its impact on auditory health, particularly in steady-state noise environments commonly found in industrial settings. One hundred million people are estimated to suffer from hearing loss due to exposure to noise [5]. Worldwide, occupational exposure to noise accounts for 3% of cases of adult hearing loss [3,6]. Generally, hearing loss will interfere with communication and hinder personal attention and cognition [7,8]. Workers exposed to steady-state noise for a long time are prone to NIHL. In special operation posts that may be exposed to different noises for a long time, hearing loss and tinnitus are common consequences of workers [9]. Steady-state noise has a dose-response relationship for people who have been exposed to it for a long time. In previous studies, NIHL predominantly manifests a high-frequency hearing loss [10–13] and typically develops most rapidly during the first 10 years of noise exposure, with subsequent progression slowing over time as cumulative damage accrues [14,15]. It is well established that the negative effects of exposure to noise are not limited to mere annoyance, but also pose a significant threat to workers’ hearing health. The extent of this risk is not only determined by the level of noise, but also by the duration of exposure. As working time exposed to noise increase, the probability of hearing damage also increases gradually. This phenomenon is particularly concerning, given that many workers in high-noise environments, such as construction sites, factories, and music venues, frequently work long hours and are often exposed to noise levels that exceed recommended limits. To prevent hearing loss, it is important for workers in high-noise environments to take appropriate measures to protect themselves from excessive noise exposure. Existing NIHL protection methods include wearing hearing protection devices, staying away from noise sources, or eliminating or reducing noise through engineering or administrative control, which is the best method for NIHL intervention [16].

For the protection of NIHL, it is necessary to carefully analyze the clinical characteristics of NIHL exposed to steady-state noise for a long time and to propose a suggestion to reduce the time of exposure to noise at a specific time. It is helpful to explore whether hearing will deteriorate significantly after a certain number of years of exposure to noise, which can guide people who have been exposed to noise to stop exposure to noise after an appropriate year. Therefore, the purpose of this study is to explore the characteristics of hearing loss exposed to noise for a long time, especially the severity of hearing loss as time changes, and to find out whether there is a time inflection point that aggravates the hearing loss the most.

## MATERIAL AND METHODS

### Participants

In December 2019, all 78 subjects working in an experimental field of an engine working who have been exposed to steady-state noise for >1 year were collected as the steady-state noise group. All subjects were asked to have no family history of hearing loss, ototoxic drug use, or nervous system disease. There were no abnormal symptoms of the hearing system, such as conscious hearing loss, tinnitus, and ear tightness before work, and they successfully passed the routine specialized physical examination for enlistment. They were also asked to be generally healthy and to have never experienced traumatic brain injury. Among them, the subjects are aged 21–55 years and have worked for periods ranging from 1.5 year to 30 years. Due to the particularity of the working environment and operations, the subjects in the collection were all male. Despite the widespread exposure to noise, none of the workers utilized hearing protection devices during their work. A control group comprising 67 male subjects was recruited from the same workplace but from non-noise-exposed job roles. The control group was matched to the noise-exposed group by age and work years to reduce potential confounding factors. All participants in the control group met the same inclusion criteria as the noise-exposed group. Both groups underwent comprehensive audiological assessments, including pure-tone audiometry (PTA) and distortion product otoacoustic emissions (DPOAE). All subjects were required to sign the informed consent form. The study was approved by the Ninth Medical Center of PLA General Hospital, Beijing, China, ethical committee and informed consent was obtained from all individual participants.

### Data collection

To evaluate workers’ exposure to steady-state noise in their working environment, noise measurements were conducted at various locations representative of the subjects’ work-places. The testing focused on engine workshops at the test site, where steady-state noise is prevalent. A total of 20 tests were performed, with measurement points strategically placed at positions reflecting the typical locations and activities of workers during their shifts. Noise levels were assessed using a calibrated sound level meter, and the measurements were carried out over an 8-hour working day to capture variations in noise exposure throughout the shift. The primary parameter measured was the A-weighted sound pressure level (L_A_), which accounts for the human ear's sensitivity to different frequencies. Each measurement point was monitored for a min. 15 min to ensure accurate and representative data collection. Following the ISO 9612 guidelines, the daily noise exposure level (L_ex, 8h_) was calculated using the formula:



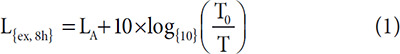



The calculation involved integrating the measured noise levels across different locations and times, considering the exposure duration at each measurement point. This approach provided a standardized assessment of the 8-hour equivalent noise exposure for workers in the study group, aligning with international standards for occupational noise assessment.

#### Hearing test method

Pure-tone audiometry was conducted using the Astera audiometer (Denmark) to evaluate the hearing function in both ears of the subjects. The testing procedure complied with the ISO 8253-1:2010 standard for audiometric testing, ensuring adherence to internationally recognized guidelines. The test was performed in a soundproof room to ensure accuracy. The measurement was carried out at the following frequencies: 0.25 kHz, 0.5 kHz, 1 kHz, 2 kHz, 3 kHz, 4 kHz, 6 kHz, and 8 kHz, tested sequentially in ascending order of frequency. The testing procedure involved presenting pure tones to each ear individually through calibrated headphones, starting at an audible intensity level. The hearing threshold at each frequency was determined using the ascending-descending method with a 5 dB step size. Specifically, the tone was initially presented at a level expected to be audible to the subject. If the subject responded, the intensity was decreased in 5 dB steps until no response was elicited. The tone was then increased in 5 dB steps until a response occurred again. This process was repeated until the lowest intensity level at which the subject responded to at least 50% of the stimuli at a given frequency was identified as the hearing threshold. For DPOAE, the Bio-Logic 580-AX2191 system (Bio-Logic, USA) was used. The procedure was carried out in accordance with established protocols aligning with ISO 389-8:2004 standards for acoustic calibration and testing methods for oto-acoustic emissions. In parameter setting, the frequency ratio f_2_/f_1_ = 1.22. For the f_2_ frequencies, measurements were conducted at 750 Hz, 1000 Hz, 2000 Hz, 3000 Hz, 4000 Hz, 6000 Hz and 8000 Hz. The stimulus intensities for the primary tones were adjusted to L_1_/L_2_ = 65/55 dB SPL, aligning with standard testing protocols to achieve reliable results. To quantify hearing loss, the difference between the distortion product (DP) amplitude and the noise floor (NF) was calculated at each f_2_ frequency. The DP-NF value was obtained by subtracting the NF level from the DP amplitude, with measurements conducted at 750 Hz, 1000 Hz, 2000 Hz, 3000 Hz, 4000 Hz, 6000 Hz, and 8000 Hz.

### Statistics

Mean (M) ± standard deviation (SD) to show the age, work time, and hearing conditions of the subjects at various frequencies. Continuous variables were analyzed using the t-test, and a p-value of <0.05 was considered statistically significant. Multivariate analysis of variance (MANOVA) was used to test the effect of exposure time to steady-state noise on hearing loss. For the main factors in evaluating hearing loss, the authors used PTA thresholds at high frequencies, 3 kHz, 4 kHz, 6 kHz, and 8 kHz, to assess the correlation between the years of work and hearing deterioration. This method was chosen to account for the simultaneous influence of multiple dependent variables, specifically the PTA and DPOAE thresholds at high frequencies (3 kHz, 4 kHz, 6 kHz, and 8 kHz). High-frequency thresholds were selected as they are particularly sensitive to early changes in hearing ability and are often used as key indicators of NIHL. Based on this, the authors selected a time point where there was a significant difference in hearing deterioration and used this time point to divide patients into 2 groups. In the analysis, the PTA and DPOAE thresholds at these frequencies were treated as dependent variables, while the exposure time to steady-state noise was treated as the independent variable. By analyzing these thresholds collectively, MANOVA enabled the authors to evaluate whether prolonged exposure time had a statistically significant effect on hearing deterioration across the high-frequency range. The assumed level of significance for all statistical analyses was set at α = 0.05. All calculations and tests were conducted using statistical software to ensure accuracy and reproducibility.

During the analysis, workers were divided into early exposure group (group A) and late exposure group (group B). This division was determined based on the most significant time intervals of hearing deterioration identified through MANOVA. The authors compared the PTA results and DPOAE results of these 2 groups. The boxplot is used to show the PTA and DPOAE at different frequencies. All statistical analyses in this report are performed using R (4.2.1).

## RESULTS

### Basic clinical and hearing characteristics

After completing the measurements, the average L_A_ in the engine workshops with steady-state noise was calculated to be 82.9 dB. This value represents the mean noise exposure level across all tested locations and provides an indication of the typical noise environment workers are subjected to during their daily activities. Variations in noise levels between different measurement points and time intervals were minimal, confirming the steady-state nature of the noise in these workshops. Based on ISO 9612 guidelines, the L_ex, 8h_ for the entire study group was calculated. The calculation considered the exposure durations at different workstations and the spatial distribution of noise levels within the working environment. As a result, the average L_ex, 8h_ for workers exposed to steady-state noise was determined to be 82.9 dB, reflecting the standardized 8-hour equivalent noise exposure for the study group. Subjects typically work in these noisy environments for 8 h daily, leading to cumulative noise exposure that may exceed permissible levels depending on specific workstation conditions.

The authors analyze the age, working years, and hearing test results of 78 noise-exposed subjects and 67 control subjects. Age and working years are shown in Table 1. The PTA thresholds are presented in Table 2, while the DPOAE results are shown in Table 3. As shown in Table 1, the average age of the noise-exposed group is M±SD 34.15±8.42 years, and the average working time is 8.81±8.04 years, while the control group has an average age of 33.40±9.14 years and an average working time of 8.97±7.14 years. There is no statistically significant difference between the 2 groups in either age (p = 0.359) or working time (p = 0.485). For PTA thresholds (Table 2), the noise-exposed group showed significantly higher hearing thresholds at most frequencies compared to the control group, particularly at higher frequencies (≥1 kHz). Statistically significant differences were observed starting at 1 kHz (p = 0.049), with the most pronounced differences at 4 kHz, 6 kHz, and 8 kHz (p < 0.001). Hearing loss in the noise-exposed group occurred primarily at high frequencies, with the worst hearing observed at 6 kHz. Similarly, the DPOAE results (Table 3) demonstrate a significant reduction in otoacoustic emissions in the noise-exposed group compared to the control group, especially at higher frequencies. Significant differences between the 2 groups were observed at 1 kHz (p = 0.019) and above, with the largest differences at 4 kHz, 6 kHz, and 8 kHz (p < 0.001). The worst DPOAE responses in the noise-exposed group occurred at 8 kHz.

**Table 1.** Demographic characteristics: age and work time in subjects working in an experimental field of an engine working, exposed to steady-state noise for >1 year, and control groups, China, December 2019

Variable	Participants (N = 145)		p
	noise-exposed group (N = 78)	control group (N = 67)	
Age [years] (M±SD)	34.15±8.42	33.40±9.14	0.359
Work time [years] (M±SD)	8.81 ±8.04	8.97±7.14	0.485

**Table 2.** Pure-tone audiometry (PTA) and distortion product otoacoustic emissions (DPOAE) threshold in subjects working in an experimental field of an engine working, exposed to steady-state noise for >1 year, and control groups, China, December 2019

Variable	Participants (N = 290)		p
	noise-exposed group (N = 156^a^)	control group (N = 134^a^)	
PTA [dB] (M±SD)			
250 Hz	16.09±5.81	13.36±5.12	0.560
500 Hz	16.51±6.11	13.58±4.57	0.249
1 kHz	17.53±7.61	12.05±4.56	0.049
2 kHz	16.86±9.76	11.12±4.41	<0.001
3 kHz	19.36±13.94	11.16±4.46	<0.001
4 kHz	24.01±18.20	12.13±4.88	<0.001
6 kHz	30.90±20.63	15.00±7.43	<0.001
8 kHz	24.29±19.73	12.39±6.39	<0.001
DPOAE [dB SPL] (M±SD)			
750 Hz	10.96±7.09	12.35±5.25	0.065
1 kHz	15.42±8.46	17.71±6.56	0.019
1.5 kHz	17.98±8.55	20.65±6.15	0.001
2 kHz	14.61±7.77	17.74±5.87	0.018
3 kHz	10.66±8.72	16.34±6.6	0.048
4 kHz	5.64±7.66	11.94±5.39	<0.001
6 kHz	2.37±6.16	8.90±5.13	<0.001
8 kHz	1.77±7.20	9.82±5.33	<0.001

a Number of ears.

**Table 3.** Comparison of pure-tone audiometry (PTA) and distortion product otoacoustic emissions (DPOAE) between the early exposure group and the late exposure group in subjects working in an experimental field of an engine working, exposed to steady-state noise for >1 year, and control groups, China, December 2019

Variable	Participants (N = 156)		p
	early exposure group (N = 74)	late exposure group (N = 82)	
PTA (ears: early exposure group N = 74, late exposure group N = 82) [dB] (M±SD)			
250 Hz	14.93±4.57	17.13±6.62	0.016
500 Hz	15.61±4.45	17.32±7.25	0.075
1 kHz	15.61±4.45	19.27±9.33	0.002
2 kHz	13.85±5.27	19.57±11.95	<0.001
3 kHz	15.27±7.35	23.05±17.23	<0.001
4 kHz	17.70±10.67	29.70±21.59	<0.001
6 kHz	22.97±13.14	38.05±23.55	<0.001
8 kHz	15.95±7.70	31.83±23.98	<0.001
DPOAE (ears: early exposure group N = 66, late exposure group N = 90) [dBSPL] (M±SD)			
750 Hz	12.77±5.70	9.64±7.75	0.004
1 kHz	17.78±7.69	13.69±8.67	0.002
1.5 kHz	20.80±7.30	15.91±8.88	<0.001
2 kHz	16.43±6.89	13.28±8.19	0.010
3 kHz	13.76±8.28	8.38±8.42	0.001
4 kHz	8.38±7.25	3.62±7.41	<0.001
6 kHz	3.53±6.41	1.52±5.89	0.048
8 kHz	2.21±6.86	1.45±7.51	0.513

Greatest disparities at 4 years for DPOAE and at 5 years for PTA following steady-state noise exposure.

**Figure 1.**
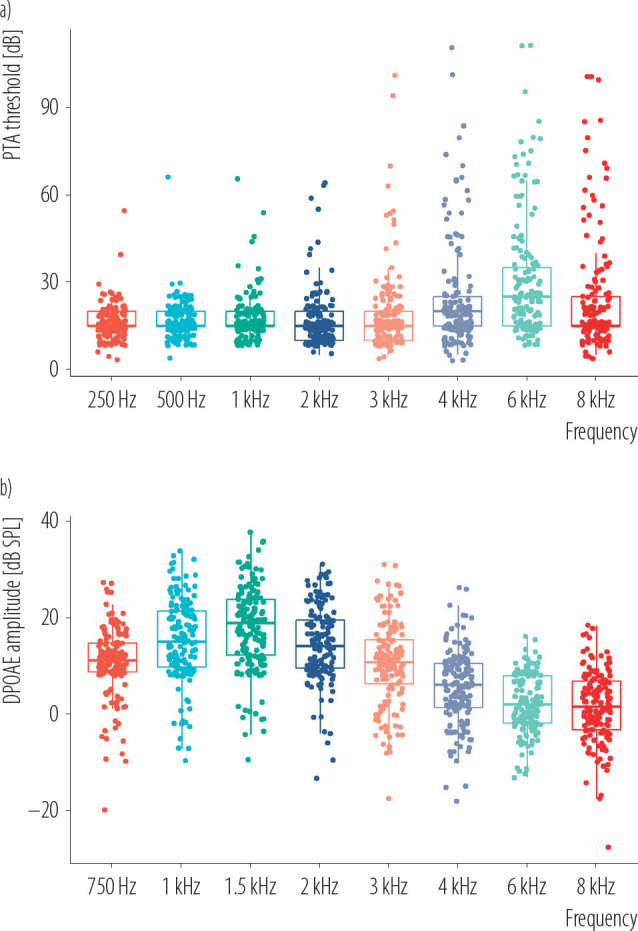
a) Pure-tone audiometry (PTA) and b) distortion product otoacoustic emissions (DPOAE) hearing threshold boxplot of noise-exposed subjects working in an experimental field of an engine working, exposed to steady-state noise for >1 year, and control groups, China, December 2019

**Figure 2.**
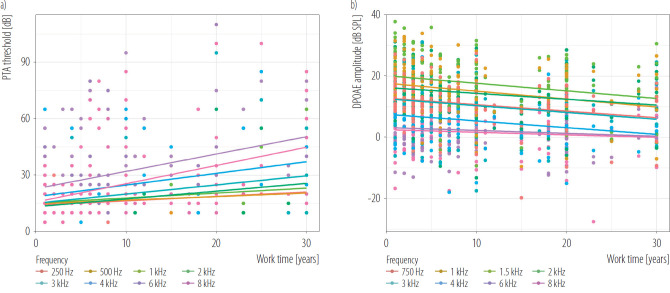
a) Pure-tone audiometry (PTA) and b) distortion product otoacoustic emissions (DPOAE) hearing threshold by the work time of the noise-exposed subjects working in an experimental field of an engine working, exposed to steady-state noise for >1 year, and control groups, China, December 2019

The boxplots in Figure 1 primarily illustrate the PTA thresholds and DPOAE results of the noise-exposed group across different frequencies. These figures provide a clear and intuitive visualization of the hearing decline observed in this group, particularly at higher frequencies. For PTA thresholds (Figure 1a), the most significant declines are evident at 3 kHz, 4 kHz, 6 kHz, and 8 kHz, with the worst hearing thresholds occurring at 6 kHz. Similarly, for DPOAE results (Figure 1b), reductions are most pronounced at frequencies of 4 kHz, 6 kHz, and 8 kHz, with the weakest otoacoustic emissions observed at 8 kHz. These visualizations emphasize the extent of high-frequency hearing impairment in the noise-exposed group compared to lower frequencies. Figure 2 show the changes in PTA and DPOAE at specific frequencies as a function of work time. At lower frequencies, there is minimal variation in hearing thresholds and otoacoustic emissions over time. However, as frequency increases, a clear trend emerges, with the degree of hearing loss becoming more pronounced at higher frequencies, highlighting a stronger correlation between extended work time and high-frequency hearing impairment.

The scatter plot shows the PTA and DPOAE threshold of all noise-exposure subjects for which work time was reported, totaling 156 ears colored by frequency.

### Analysis of the time inflection point of early exposure and late exposure at the highest hearing deterioration

**Figure 3.**
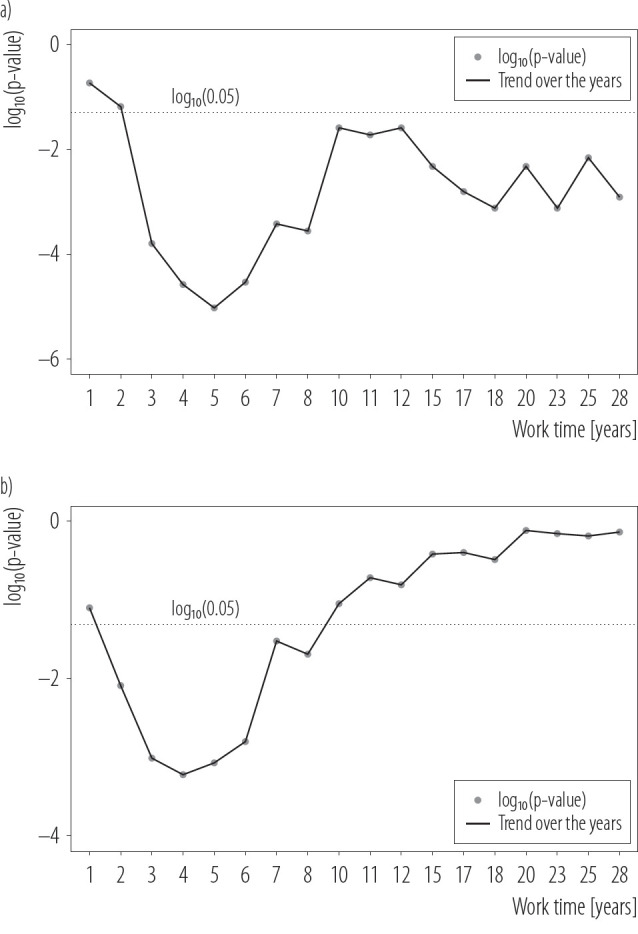
The temporal inflection point at which the high frequencies (3 kHz, 4 kHz, 6 kHz and 8 kHz) of the early exposure group and the late exposure group, separated from among subjects working in an experimental field of an engine operation exposed to steady-state noise for >1 year, showed the greatest discrepancies, assessed using MANOVA, China, December 2019

The authors analyze the relationship between the time inflection point of the most serious hearing deterioration and the high-frequency NIHL (3 kHz, 4 kHz, 6 kHz and 8 kHz) through MANOVA (Figure 3). With the increase in exposure time to steady-state noise, hearing loss at high frequencies is gradually increasing. One-way MANOVA revealed a significant multivariate main effect of the time inflection point, 5 years, on hearing loss, as shown by 3 kHz, 4 kHz, 6 kHz, and 8 kHz in the PTA threshold. In the fourth year, it was the high-frequency hearing loss measured by DPOAE that showed the time inflection point with the largest difference between the early exposure group and the late exposure group. And after 10 years, the difference in DPOAE amplitudes between the 2 groups was not statistically significant by comparing with log_10_ p-value in Figure 3. It can also be seen in Figure 3 that DPOAE can show the time inflection point of the most serious hearing deterioration 1 year earlier than PTA.

The difference was most pronounced in year 5 (p = 9.46 × 10^−6^) under pure-tone audiometry (PTA) (Figure 3a), and in year 4 (p = 6.01 × 10^−4^) under distortion product otoacoustic emissions (DPOAE) (Figure 3b). There were no significant differences in DPOAE measurements after 10 years of exposure (Figure 3b).

### Comparison between the 2 groups according to time inflection with the greatest hearing deterioration

**Figure 4.**
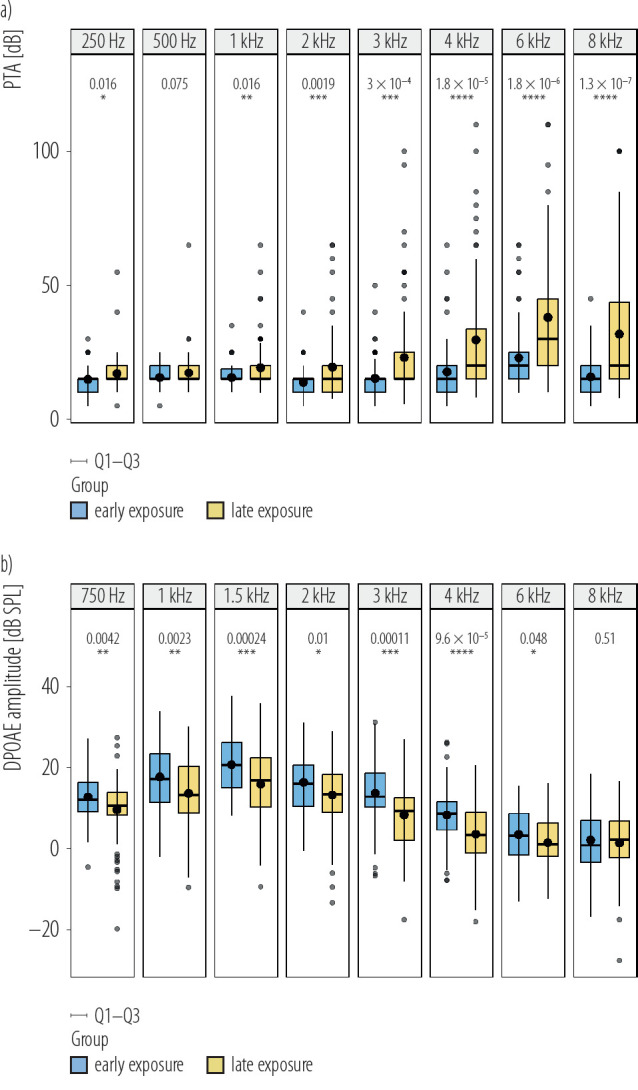
Comparison of hearing loss of the early exposure group and the late exposure group, separated from among subjects working in an experimental field of an engine operation exposed to steady-state noise for >1 year, under a) pure-tone audiometry (PTA) and b) distortion product otoacoustic emissions (DPOAE), China, December 2019

Table 4 shows the comparison of hearing between the early exposure group (group A) and the late exposure group (group B). Through PTA, 37 of 78 subjects (47.4%) in noise-exposed group were classified as group A and 41 (52.6%) as group B. Through DPOAE, 33 of 78 (42.3%) subjects were classified as group A and 45 (57.7%) as group B. There were significant differences in hearing loss between the 2 groups (Figure 4). Figure 4 visualize the results of 2 groups of subjects in PTA and DPOAE. However, in PTA, at the frequency of 500 Hz, the loss of group A and group B was not statistically significant. It shows that in the 500 Hz, PTA as a subjective test has not caused much difference in damage. In DPOAE results, at the frequency of 8 kHz, there was no statistically significant loss between group A and group B, so it may indicate that at 8 kHz, DPOAE detects similar irreversible outer hair cell (OHC) damage. The main difference between group A and group B is still at the hearing threshold of 3 kHz, 4 kHz, and 6 kHz. The decrease in high-frequency hearing threshold is also a major feature of NIHL.

* p < 0.05; ** p < 0.01; *** p < 0.001; **** p < 0.0001.

## DISCUSSION

Hearing loss caused by workplace noise exposure is a major health problem around the world. Noise-induced hearing loss is a multifactorial condition influenced by both the intensity and duration of noise exposure. High noise levels, such as steady-state noise >85 dB over extended periods like 1 year, significantly increase the risk of NIHL by causing apoptosis of cochlear hair cells, which do not regenerate once damaged [7,17]. Occupational groups, such as construction workers and factory employees, face a particularly high risk due to long working hours and frequent exposure to noise [18,19]. Prolonged noise exposure also accelerates age-related hearing loss, leading to premature aging of the inner ear [20,21]. Moreover, the extent of NIHL risk is not only determined by the level of noise but also by the duration of exposure, as longer working hours in high-noise environments steadily increase the likelihood of hearing damage. Workers in industries such as construction, manufacturing, and music venues often experience this cumulative risk. Although the mechanisms of NIHL are complex and not fully understood, it is widely accepted that exposure to loud noise damages the delicate hair cells in the cochlea, impairing their ability to transmit sound signals to the brain.

The risk of hearing deterioration in noisy work environments is a serious concern that requires attention from both workers and employers. Noise-induced hearing loss has been reported to be significantly related to the increased risk of industrial injuries and non-auditory issues, such as mood disorders, sleep disturbances, and cardiovascular diseases [7,22,23]. Given that hearing loss becomes more severe with prolonged noise exposure and aging, it is essential to identify a critical time inflection point where hearing deterioration is most pronounced. Determining this time frame could help establish guidelines for limiting exposure duration, reminding workers to reduce contact with noise sources or adopt effective hearing protection devices. This proactive approach is crucial for mitigating both auditory and non-auditory sequelae associated with NIHL and ensuring better occupational health outcomes. In the study, the authors used PTA and DPOAE to assess hearing loss in both subjects exposed to steady-state noise and those in the control group. Compared to the control group, hearing loss in the noise-exposed group was predominantly concentrated in the high-frequency ranges. To better characterize the features of hearing loss specifically in the noise-exposed group, the authors focused their analysis on individuals with confirmed hearing impairment due to noise exposure. To analyze high-frequency hearing loss, the authors applied MANOVA (multivariate analysis of variance), which allowed them to simultaneously assess the effects of noise exposure duration on hearing thresholds across multiple frequencies (3 kHz, 4 kHz, 6 kHz, and 8 kHz). In this analysis, the hearing thresholds at these 4 frequencies were treated as the dependent variables, while the duration of noise exposure was categorized as the independent variable. Specifically, the exposure durations were divided into binary groups for each year of exposure (e.g., >1 year vs. ≤1 year, >2 years vs. ≤2 years, and so on). This approach enabled the authors to identify the year with the smallest p-value as the time inflection point, which represents the year in which the most significant difference in hearing thresholds emerged between the shorter and longer exposure groups. By leveraging this method, the authors were able to pinpoint the critical time at which hearing deterioration becomes particularly pronounced. The use of MANOVA is especially advantageous in this context, as it accounts for the interdependencies among the 4 frequency thresholds, providing a more comprehensive understanding of high-frequency hearing loss patterns over time. The authors’ analysis revealed that with increasing exposure duration, high-frequency hearing loss became progressively more significant, with the largest differences occurring around the identified time inflection point. Specifically, DPOAE assessments showed the most significant change in high-frequency hearing loss at the 4-year exposure mark, while PTA assessments revealed the greatest difference at 5 years of exposure. This finding highlights the earlier detection capability of DPOAE compared to PTA in identifying the onset of significant hearing deterioration due to noise exposure.

In humans, DPOAE has been widely used for the early and differential diagnosis of cochlear OHC injury, which is a common cause of NIHL. Distortion product otoacoustic emissions measures the sound signals produced by the OHCs in response to 2 simultaneous tones, and any deviation from the normal range indicates potential damage or dysfunction of the OHCs. As a non-invasive and objective test, DPOAE is particularly useful for screening the hearing function in young children and individuals who may not be able to provide reliable feedback. Moreover, DPOAE can detect OHC damage before subjective hearing loss becomes apparent, which makes it an effective tool for early detection [24,25]. In the study, the authors observed a significant decrease in the DPOAE amplitude with increasing exposure duration. These findings are consistent with the PTA results, where the prevalence of high-frequency hearing loss increased with prolonged noise exposure. Both methods indicate that prolonged exposure to environmental steady-state noise significantly impacts high-frequency hearing, with the greatest changes occurring over time. Cumulative hearing loss, resulting from long-term exposure, leads to a gradual decline in hearing sensitivity. Despite the long-standing recognition of noise's detrimental effects on hearing, there remains uncertainty regarding the optimal approach for diagnosing cumulative hearing loss in clinical practice. Additionally, the inflection point at which hearing loss becomes clinically significant and requires intervention is still a subject of ongoing research. The authors’ analysis of the time inflection point for the most significant hearing impairment revealed that DPOAE could detect the critical point of hearing deterioration 1 year earlier than PTA. This finding underscores the ability of DPOAE to identify the threshold at which hearing deterioration due to NIHL occurs, offering a lead time of 1 year compared to PTA measurements. This earlier detection capability is consistent with the understanding that noise-induced OHC dysfunction may precede subjective hearing loss. Increasing evidence suggests that repeated or prolonged noise exposure can lead to structural changes in the inner ear, contributing to hidden hearing loss (HHL) that precedes detectable hearing loss [20]. In the authors’ study, the DPOAE results showed that the inflection point of hearing deterioration was earlier than the PTA results, confirming that DPOAE could detect OHC dysfunction 1 year before the subjective detection of hearing loss by PTA. This is also supported by the study by Hoben et al. [26], which identified OHC dysfunction as a key factor in HHL. Distortion product otoacoustic emissions has been widely recognized as an important tool for detecting subtle cochlear dysfunction, especially in the early stages of NIHL. For instance, studies have shown that DPOAE can identify changes in cochlear function even when conventional audiometric thresholds remain normal, making it particularly valuable for early detection. A study by Bruce et al. [27] demonstrated that DPOAE can detect cochlear damage in individuals with normal hearing thresholds, offering insight into HHL before the onset of measurable changes in pure tone audiometry. Additionally, DPOAE has been shown to be more sensitive in detecting early-stage cochlear damage in noisy environments, where traditional audiometric tests may fail to capture subtle dysfunction. This ability to detect hidden cochlear damage underscores the importance of DPOAE in diagnosing NIHL before it becomes clinically evident [28,29].

To further illustrate this, the authors examined the p-value curves for PTA and DPOAE across different exposure durations. In the PTA analysis, the p-value was >0.05 during the first 2 years of noise exposure, indicating no significant difference in hearing thresholds between individuals with ≤1 year and >1 year or ≤2 years and >2 years of exposure. After 1 year of exposure, the p-value gradually decreased, with the smallest p-value observed at the 5-year mark, indicating the most significant difference in hearing thresholds between groups with ≤5 years and >5 years of exposure. However, starting from the sixth year, the p-value began to increase again, suggesting that the difference in PTA thresholds between shorter and longer exposure durations became less significant. Similarly, in the DPOAE analysis, the p-value showed a gradual decrease after 1 year of exposure, reaching its lowest point at 4 years of exposure. This indicates that the most significant difference in high-frequency DPOAE results occurred between individuals exposed to noise for ≤4 years and those exposed for >4 years. Beyond the 5-year exposure mark, the p-value began to rise. In the authors’ comparison between the early and late exposure groups, the authors observed no significant difference in DPOAE at 8 kHz in the fourth year, suggesting possible OHC damage occurring before 4 years of exposure. By the fifth year, PTA results showed no significant subjective hearing loss difference at 500 Hz between the 2 groups, indicating less severe progression at lower frequencies. While slight differences existed in 500 Hz frequency between the groups, they were not statistically significant in the study population.

This study has limitations, including its retrospective design and small sample size. Future large-scale prospective cohort studies with long-term follow-up are warranted to validate and augment the authors’ findings. Further investigation is required to delineate the optimal time inflection point for hearing loss due to prolonged noise exposure. Nonetheless, this study is the first to propose such a point for high-frequency hearing loss deterioration in both PTA and DPOAE among noise-exposed workers.

In summary, identifying the time inflection point for hearing deterioration aids in understanding the impact of long-term steady-state noise exposure on hearing loss and provides valuable guidance for hearing protection strategies in noise-exposed individuals.

## CONCLUSIONS

In conclusion, a pronounced exacerbation of NIHL at high frequencies correlates with prolonged noise exposure. After 4 years of exposure, DPOAE assessments revealed the most significant difference in high-frequency hearing loss between individuals exposed to noise for ≤4 years and >4 years, indicating the inflection point of hearing deterioration. Similarly, PTA assessments showed the greatest difference in high-frequency hearing loss between individuals exposed to noise for ≤5 years and >5 years, marking the point of maximal change in PTA and representing the inflection point of hearing impairment. Distortion product otoacoustic emissions also demonstrated an ability to detect worsening of hearing loss approx. 1 year earlier than subjective perception, highlighting its potential for early detection of inner ear damage in NIHL. Addressing strategies for the prevention and mitigation of long-term noise-induced hearing deterioration emerges as a critical area necessitating further active research in the future.
